# Direct Affinity Purification of Long‐Acting PASylated Proteins with Therapeutic Potential Using L‐Prolinamide for Mild Elution

**DOI:** 10.1002/anie.202200079

**Published:** 2022-04-27

**Authors:** Jonas Schilz, Charlotte Clement, Franziska Greiner, Arne Skerra

**Affiliations:** ^1^ Lehrstuhl für Biologische Chemie Technische Universität München Emil-Erlenmeyer-Forum 5 85354 Freising (Weihenstephan) Germany

**Keywords:** Affinity Chromatography, Antibody, L-Prolinamide, PASylation, Protein Purification

## Abstract

Both insufficient plasma half‐life (circulation for only few hours or less) and laborious downstream purification can be bottleneck for biological drug development. We report a novel strategy for the efficient and gentle affinity purification of pharmacologically relevant proteins modified by PASylation for prolonged action in vivo. We previously described antibodies specific for Pro/Ala‐rich sequences (PAS) covering a range of binding characteristics. Our present approach relies on a chromatography matrix functionalized with a low‐affinity PAS‐specific antibody Fab fragment for specific adsorption of the PASylated protein from a macromolecular mixture. With the complete absence of hydrophobic/aromatic or ionic groups in the PAS sequence epitope, binding is mediated by Van der Waals contacts and distinct hydrogen bonds only. Surprisingly, selective competitive elution is achieved by application of the highly soluble and biologically inactive imino acid derivative L‐prolinamide. Based on the specific but strongly dynamic biomolecular interaction, our procedure allows the direct one‐step purification of PASylated proteins from a cell extract or culture supernatant while avoiding harsh elution conditions as they are often needed for conventional affinity chromatography.

## Graphical abstract

PASylation of pharmacologically active proteins or peptides drastically expands their hydrodynamic volume and, thus, retards renal filtration, which results in prolonged in vivo half‐life. We have now developed an antibody‐based affinity chromatography that permits the one‐step purification of PASylated molecules in the native state using L‐prolinamide as mild elution reagent.

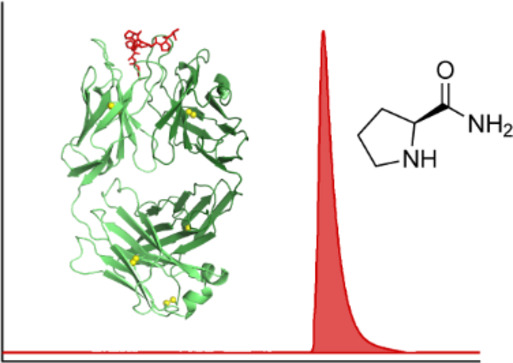

Biotherapeutics, in particular therapeutic proteins and peptides, have become the fastest growing sector in the pharmaceutical industry^[1]^ and continue to pose challenges for innovators as well as manufacturers to stay competitive. Among these, the rapid and efficient purification of recombinant proteins remains an issue both at the drug discovery or optimization stage and during development as well as manufacturing, with downstream purification accounting for a significant proportion of the total production cost.^[2]^ Affinity chromatography, which involves the genetic fusion of the pharmacologically active protein with a dedicated affinity tag, e.g. the His_6_‐, Flag‐ or *Strep*‐tag,^[3]^ is the most common method today for selective protein purification both in research laboratories and in the biotechnological industry. Corresponding fusion proteins are produced by recombinant gene expression in a microbial or mammalian host cell as well as preparation of a suitable cell extract or culture supernatant, which is followed by chromatographic purification via the specific interaction with an appropriately functionalized solid matrix. However, despite wide use in research, potential immunogenicity and/or the need for subsequent proteolytic removal of the affinity tag^[4]^ compromise the benefits and hamper the application for biopharmaceutical development and drug manufacturing.^[5]^ Consequently, there is a strong desire for tag‐free affinity purification techniques that are specific but still amenable to a wide group of relevant biological drug candidates. One prominent example is the protein A affinity chromatography for the purification of recombinant or monoclonal antibodies (MAbs) which is based on the interaction with a conserved part of the antibody molecule such that no additional tag is needed.^[6]^ However, the harsh acidic conditions required for the quantitative elution from the protein A chromatography matrix can lead to chemical protein modification and undesired product inhomogeneity.^[7]^

Apart from the currently expanding area of therapeutic MAbs, classical protein and peptide drugs, including growth hormones, cytokines, incretins, or their antagonists, as well as enzymes, offer attractive opportunities for the development of improved versions of established biopharmaceuticals, so‐called biobetters.^[8]^ In contrast to MAbs, these biopharmaceuticals do not provide for a generic purification scheme and they also suffer from a short circulation both in patients and, even more pronounced, in small animal models. Therefore, the use of artificial structurally disordered fusion polypeptides to extend the half‐life and boost the in vivo activity of protein and peptide drug candidates has attracted considerable interest in pharmaceutical biotechnology.^[9]^ In particular, the conjugation with long polypeptides comprising the three small natural L‐amino acids Pro, Ala and/or Ser, known as PASylation® technology, dramatically expands the hydrodynamic volume of biopharmaceuticals and strongly prolongs their plasma half‐life by retarding renal filtration, depending on the length of the PAS chain (typically in the range of 200–800 residues).^[10]^ The poor immunogenicity of such feature‐less PAS biopolymers was evident in multiple preclinical studies involving repeated administration of various PASylated proteins at high doses, where no PAS‐specific antibody response was observed.^[11]^ However, by applying a tenacious immunization scheme, involving a PAS peptide conjugate with a strongly immunogenic carrier protein and repeated boosting in mice, we recently succeeded in raising a few MAbs that recognize PAS sequences with pronounced specificity.^[11a]^ While the few high‐avidity MAbs that were identified in this preceding study provide valuable tools for diagnostic assay development on the route to clinical translation,^[11b]^ those MAbs with intermediate affinity and, consequently, faster association/dissociation kinetics appeared attractive as binding partners for affinity purification purposes. Thus, we sought to combine the polypeptide tag as part of all PASylated proteins with the monovalent antigen‐binding fragment (Fab) of a less tightly interacting anti‐PAS MAb in order to functionalize a cognate affinity matrix.

Based on the previously published biochemical characterization of MAbs, and corresponding Fabs, that were specifically raised against a set of PAS sequences,^[11a]^ we decided to employ the recombinant anti‐PAS Fab 1.2 (Scheme 1) as a binding partner to prepare a novel affinity support. Fab 1.2 exhibits rather modest affinity towards the PAS#1 sequence, with a monovalent *K*_D_ value of 3.7±0.2 μM and a relatively fast dissociation kinetics (τ_1/2_=6.4 s). This is similar to the rate of dissociation determined for the interaction between the His_6_‐tag and Ni^2+^•NTA.^[12]^ Thus, a sharp elution of the affinity‐captured protein of interest can be expected. A moderate to weak binding interaction becomes even more important in the context of a potential avidity effect when dealing with proteins that are fused with a long PAS polypeptide, e.g. PAS#1(200). In such a case, 10 copies of the repetitive 20mer PAS#1 sequence motif are present (Scheme S1),^[9a]^ which leads to much enhanced binding activity for the corresponding bivalent MAb.^[11a]^

**Scheme 1:**
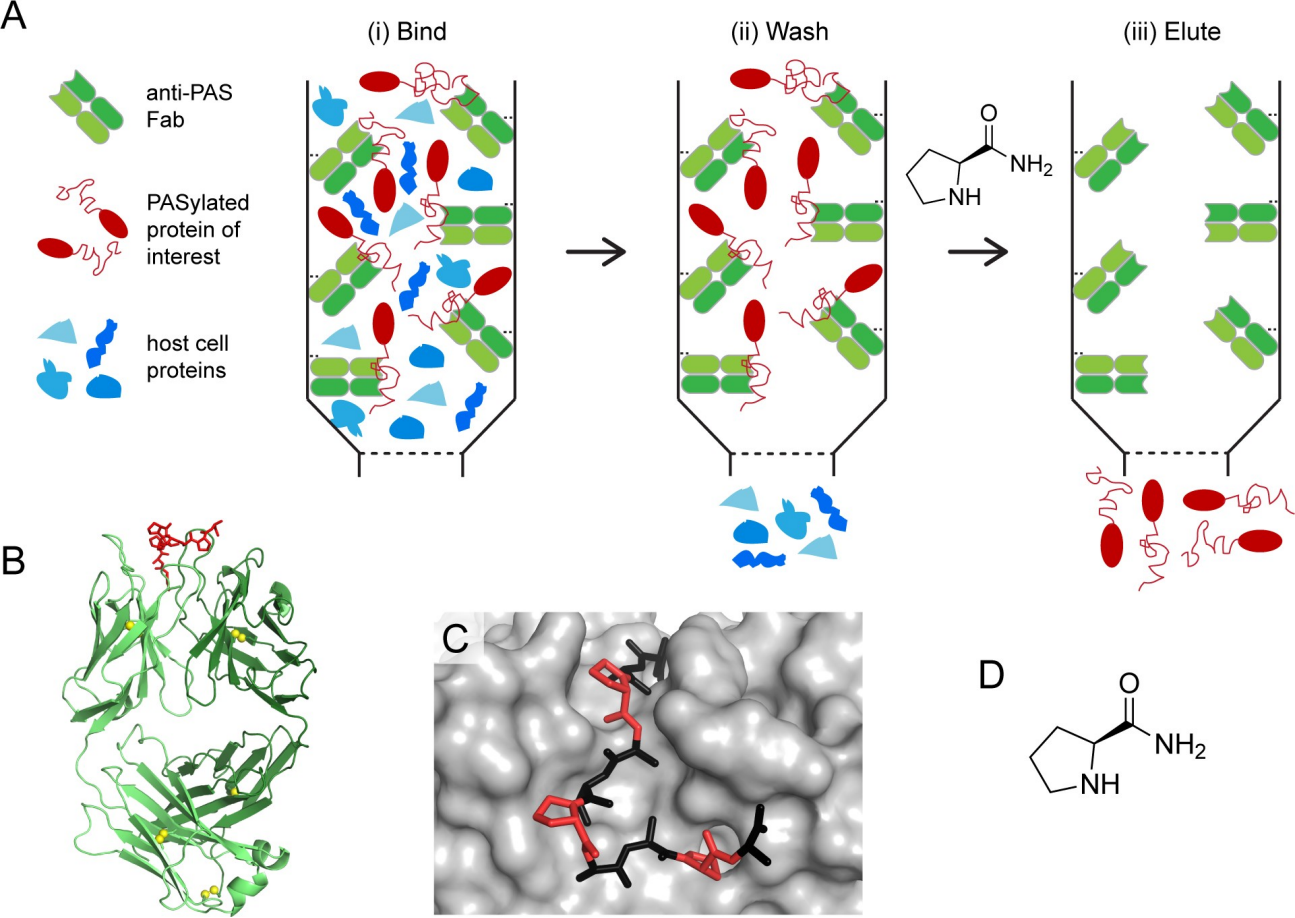
Principle of the affinity purification of PASylated proteins using a column with the immobilised anti‐PAS Fab 1.2. A) Schematic illustration of the one‐step PAS affinity purification: (i) application of the cell extract containing the PASylated protein of interest, (ii) column washing with a physiological running buffer and (iii) elution of the PASylated protein by applying a 1 M solution of L‐prolinamide in running buffer at pH 8. B) Crystal structure (PDB ID: 7O31) of Fab 1.2 (light chain: light green; heavy chain: dark green; for the amino acid sequences, see Scheme S1) in complex with its PAS#1 epitope peptide (red). C) Top view on the paratope of Fab 1.2 with the bound minimal PAS epitope peptide shown as sticks and L‐prolinamide moieties therein highlighted red. D) Chemical structure of L‐prolinamide.

The next question was to identify a suitable mild buffer that efficiently competes with the affinity interaction of the PASylated protein and enables its quick elution from the column. To this end, we set up small scale binding and elution experiments using a 20 μL volume of *N*‐hydroxysuccinimide (NHS)‐activated Sepharose beads charged with the covalently immobilized recombinant Fab. Subsequently, binding and elution of the PASylated target protein was monitored by SDS‐PAGE analysis of the supernatant after resuspension of the beads and gentle centrifugation in a small reaction vessel, involving three steps: (i) incubation of the Fab matrix with a solution of the PASylated protein, (ii) washing of the beads with buffer and (iii) incubation with the elution agent. Following this approach, and employing the pre‐purified PASylated enhanced green fluorescent protein (eGFP) as a model analyte, we tested various elution conditions: low or high pH, elevated ionic strength, chaotropic salts, denaturants, organic solvents, polyethylene‐glycol as well as several small molecule competitors.

Buffers with elevated ionic strength did not effect elution, in agreement with our findings from the crystal structure of the corresponding Fab•PAS peptide complex where no salt‐bridges were observed due to the complete absence of charged amino acids in the PAS sequence. Low pH, denaturing buffers (1–4 M urea, 1–2 M Gdn•HCl, 0.5–3 % (w/v) SDS) and certain small molecule competitors showed more promising results. Hence, we focused on the latter as a less detrimental influence on protein integrity was anticipated. Indeed, among the specific small molecule competitors the N‐terminally blocked minimal PAS epitope peptide—amino acid sequence: Pga‐APASPAAPA (Pga: pyroglutamyl)—led to efficient elution of the PASylated eGFP at 2 mM concentration. However, this approach was not further elaborated due to the high cost of synthetic peptides which precludes wider application, in particular at preparative scale. Suprisingly, a 1 M solution of the single derivatized amino acid L‐prolinamide appeared to be equally effective in disrupting the binding interaction, evoking quantitative elution of the bound PASylated protein.

To mimic a more typical research application using an automated chromatography system, a HiTrap NHS‐activated Sepharose column with 1 ml bed volume was covalently charged with 5 mg of the recombinant anti‐PAS Fab 1.2. Using again *Strep*II‐eGFP‐PAS#1(200) as test protein, pre‐purified from an *E. coli* total cell extract via Strep‐Tactin affinity chromatography,^[13]^ we found that the PASylated protein is tightly captured on the PAS affinity column whereas elution occurs in a sharp peak after applying 1 M L‐prolinamide in a mild running buffer (100 mM Tris/HCl pH 8.0, 150 mM NaCl, 1 mM EDTA) as mobile phase (Figure 1A).

**Figure 1:**
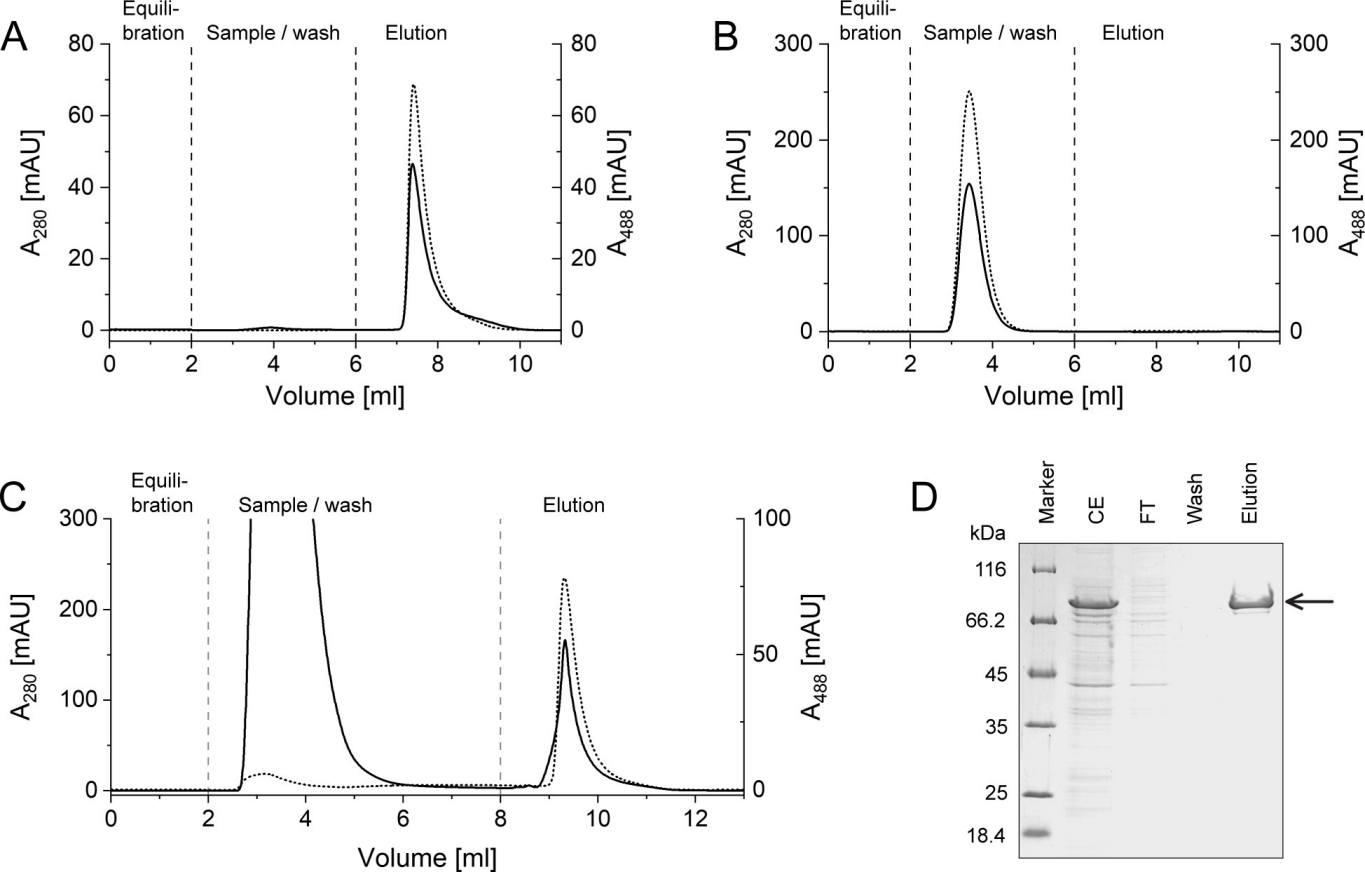
Chromatograms illustrating PAS affinity chromatography runs with A) pre‐purified *Strep*II‐eGFP‐PAS#1(200), B) pre‐purified, non‐PASylated *Strep*II‐eGFP (negative control) and C) *Strep*II‐eGFP‐PAS#1(200) from an *E. coli* whole cell extract. Monitoring of UV absorption is shown for wavelengths of 280 nm indicative of protein Tyr/Trp absorption (solid black line, corrected for background absorbance) and of 488 nm to detect the eGFP fluorophore (dotted line). D) SDS‐PAGE of representative samples from (C) including whole cell extract (CE), flow‐through (FT), washing fraction and the affinity‐purified *Strep*II‐eGFP‐PAS#1(200) protein in the prolinamide elution fraction (arrow).

In the next step, the PAS affinity chromatography was employed for the single‐step purification of a PASylated protein directly from the whole cell extract of *E. coli*. As result, the *Strep*II‐eGFP‐PAS#1(200) test protein was recovered from the column in a highly pure form while the host cell proteins where completely washed away in the flow‐through (Figure 1C,D). As a control experiment, the non‐PASylated *Strep*II‐eGFP was subjected to the chromatography, where no binding to the column was observed (Figure 1B).

Finally, practical applicability of the PAS affinity chromatography was demonstrated for two therapeutically relevant PASylated proteins (Figure 2): a PASylated cytokine which has shown superior efficacy in a mouse model of peritonitis, PAS#1(800)‐IL1Ra,^[14]^ and a PASylated Anticalin that inhibits aggregation and prevents Aβ cytotoxicity in neuronal cell culture, H1GA‐PAS#1(200)‐His_6_.^[15]^ In each case, purification of the recombinant protein from either the periplasmic or the whole cell fraction of *E. coli* was achieved by means of PAS affinity chromatography in one step.

**Figure 2:**
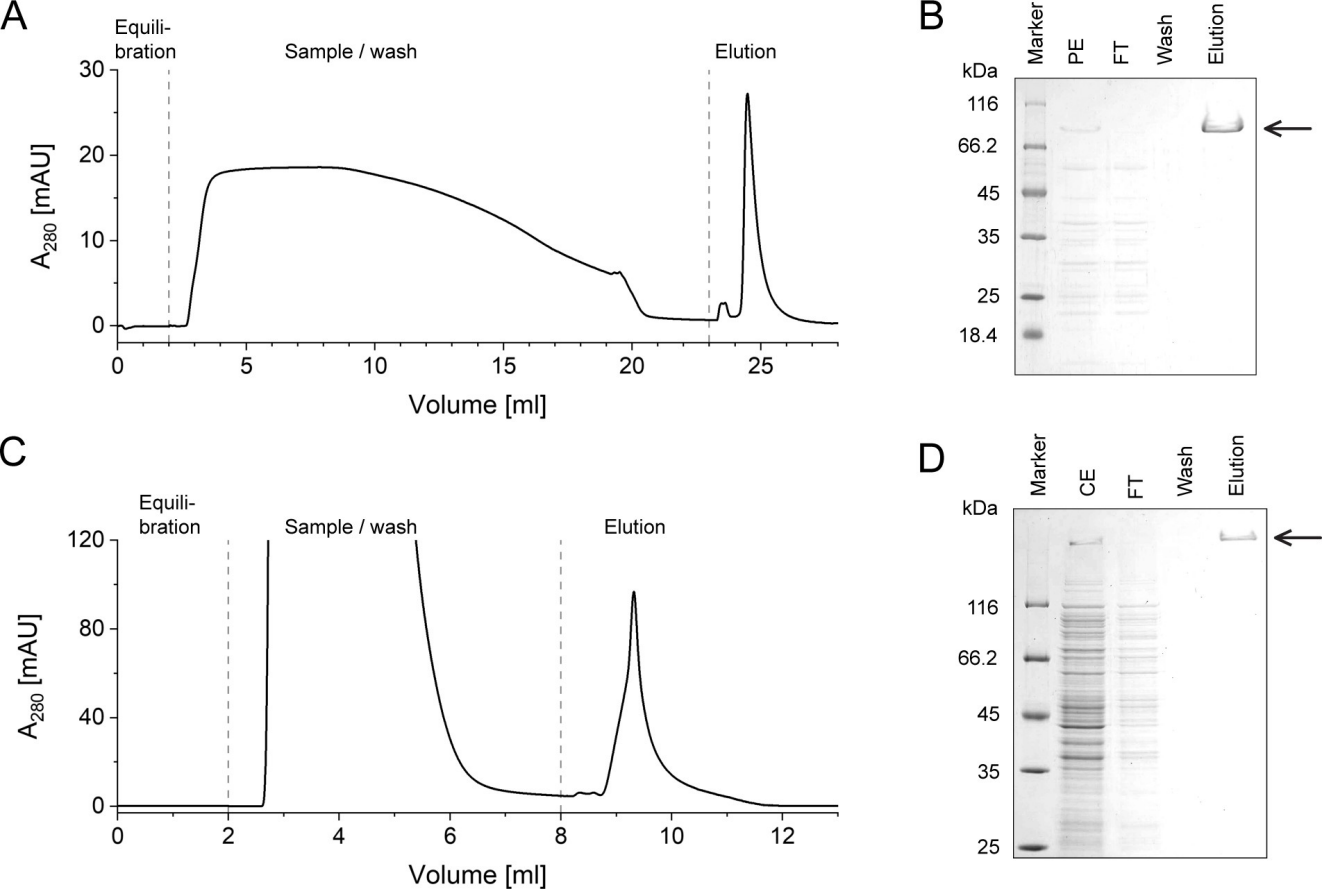
Chromatograms (A,C) and SDS‐PAGE (B,D) documenting the one‐step PAS affinity purification of H1GA‐PAS#1(200)‐His_6_ from the periplasmic extract (A,B) and of PAS#1(800)‐IL1Ra from the whole cell extract (C,D) of *E. coli*. Arrows indicate the bands corresponding to both PASylated proteins.

Apart from the high specificity of the anti‐PAS Fab, hence proven under chromatography conditions, the PAS affinity matrix appeared to be remarkably robust and the purification process was highly reproducible during multiple use. This was demonstrated by performing 20 repeated purification runs on the same 1 mL PAS affinity column under identical conditions, each time loading 500 μg *Strep*II‐eGFP‐PAS#1(200) as sample and applying 3 ml of 1 M L‐prolinamide in running buffer for elution. The 20 superimposed chromatograms showed perfect match (Figure S1D), with standard deviations of merely 1.8 % for the peak area and 0.8 % for the peak height, thus indicating little or no variation in binding capacity and elution behaviour of the PAS chromatography matrix.

Furthermore, the integrity of the PASylated protein after the PAS affinity purification was investigated by analytical size exclusion chromatography (SEC). Comparison of the resulting peaks in SEC from three different protein samples before and after the affinity purification each demonstrated a homogeneous peak without signs of protein oligomerization or aggregation (Figure S1A–C). Thus, the PAS affinity chromatography allows protein recovery in the native state, obviously owing to the avoidance of harsh elution conditions such as low pH. After appropriate dilution, the isolated PASylated protein can be directly applied to functional assays, for example ELISA or SPR measurements, cell culture assays and the like. If necessary, the L‐prolinamide can be easily removed by dialysis or with the help of a desalting column.

Using three different PASylated proteins, we have demonstrated that the newly developed anti‐PAS chromatography matrix allows the one‐step purification of proteins modified by PASylation technology without the need for additional affinity tags. Due to the pronounced specificity, albeit moderate affinity, of the anti‐PAS Fab that was chosen as binding partner^[11a]^ bacterial cell proteins were efficiently removed while only the target protein bound to the chromatography matrix via its PAS moiety (Scheme 1A). Combined with the use of L‐prolinamide as a highly soluble and biochemically rather inert substance^[16]^ for competitive elution, PAS affinity chromatography offers a mild procedure for the rapid and facile isolation of many different PASylated proteins.^[10]^

Apart from the quest for selective binding of the molecule of interest, a common challenge in affinity chromatography is the search for elution conditions that neither affect the integrity of the purified biomolecule nor harm the affinity matrix, thus enabling repeated purification cycles. However, many well established affinity chromatography procedures, including protein A and protein G chromatography for antibody purification as well as MAb‐based affinity purification of proteins fused with an epitope tag (e.g. the anti‐myc MAb 9E10 or anti‐FLAG Mab M1), or S‐tag chromatography, involve acid pH and/or high ionic strength for elution, for example 0.2 M citrate pH 2.^[3]^ Such harsh conditions can disrupt the native fold, lead to aggregate formation or even provoke chemical modification of the purified protein, in particular Asn/Gln deamidation.[^7b^, ^7d^, ^17^]

In contrast, the concept of the biochemically mild PAS affinity chromatography takes advantage of structural insights into the antibody/epitope interaction.^[11a]^ During our previous X‐ray crystallographic analysis, we found that hydrogen bond networks involving the peptide backbone (Figure S2) as well as multiple Van der Waals contacts arising from intimate shape complementarity dominate the interactions between the fully uncharged PAS polypeptide and its cognate antibodies. Thus, it was not surprising that elution conditions based on elevated ionic strength or pH shift did not lead to success whereas, in principle, a competitive ligand should be capable of interfering with these biomolecular interactions. Unexpectedly, the small molecule L‐prolinamide turned out to offer this function. Notably, this simple ligand does not only exhibit the proline moiety that constitutes a major repeated building block of PAS sequences,^[9a]^ but also its carboxamide part mimics the peptide bond with the subsequent amino acid residue, usually L‐Ala (Scheme 1C,D). This carboxamide modification abolishes a negative charge, which is in line with the lack of electrostatic interactions as seen in the native Fab•PAS complex. For comparison, no efficient elution from the PAS affinity column was observed with solutions of the zwitterionic L‐proline (at pH 8) up to 2 M concentration. Besides, L‐prolinamide, a well‐known protein‐stabilizing osmolyte compound in many organisms,^[18]^ is a harmless substance and readily available from various suppliers at a price (≈0.5 € per gram) comparable to other chemicals commonly used in therapeutic protein production (e.g., pure Gdn•HCl) and, therefore, even suitable at larger purification scale. In addition, the binding interaction between L‐prolinamide itself and the chromatography matrix is negligible such that regeneration of the PAS affinity column is simply achieved by washing with 2–3 bed volumes of running buffer. Nevertheless, apart from the elution reagent the cost and the binding capacity of the affinity resin, as well as additional steps for DNA and endotoxin removal, need to be considered prior to industrial adaptation.

In conclusion, the PAS affinity chromatography described herein has proven applicability to N‐ and C‐terminally PASylated proteins carrying PAS tags comprising 200–800 residues by taking advantage of an anti‐PAS Fab with high specificity in conjunction with fast association/dissociation kinetics. On the other hand, an anti‐PAS Fab with higher intrinsic affinity^[11a]^ might be advantageous for the purification of fusion proteins with shorter PAS tags. Thus, beyond their utility for bioanalytical and diagnostic assays in the context of the preclinical and clinical development of PASylated biopharmaceuticals, our recently generated anti‐PAS MAbs^[11a]^ considerably facilitate the routine affinity purification of corresponding drug candidates in a native state, thus obviating the need for any additional—and potentially immunogenic—purification tag.

## Experimental Section

Full description of the experimental details is provided in the Supporting Information for this article.

## Supporting information

As a service to our authors and readers, this journal provides supporting information supplied by the authors. Such materials are peer reviewed and may be re‐organized for online delivery, but are not copy‐edited or typeset. Technical support issues arising from supporting information (other than missing files) should be addressed to the authors.

Supporting Information

## Data Availability

The data that support the findings of this study are available in the Supporting Information of this article.
