# Supplementary material for: Direct Affinity Purification of Long‐Acting PASylated Proteins with Therapeutic Potential Using L‐Prolinamide for Mild Elution
Source: Angew Chem Int Ed Engl. 2022 Apr 27;61(25):e202200079. doi: 10.1002/anie.202200079 (PMC9320812; doi:10.1002/anie.202200079)
Supplement: Supplementary file 1 — Supporting Information [file ANIE-61-0-s001.pdf]

Angewandte Chemie
Eine Zeitschrift der Gesellschaft Deutscher Chemiker

Supporting Information

Direct Affinity Purification of Long-Acting PASylated Proteins with Therapeutic Potential Using L-Prolinamide for Mild Elution

*J. Schilz, C. Clement, F. Greiner, A. Skerra**

Supporting Methods

E. coli production and purification of Fab fragments

The anti-PAS Fab 1.2 was expressed and purified as previously described.[1] In brief, pASK88 derivatives harboring the corresponding V-genes were used to express the chimeric Fab fragment with human IgG1/κ domains and a His_6 tag fused to the heavy chain using the *E. coli* strain KS272[2] via 8 liter bench top fermentation in line with published procedures.[3] The recombinant Fab was purified to homogeneity from the periplasmic bacterial extract via immobilized metal ion affinity chromatography (IMAC) followed by cation exchange chromatography (CEX).

E. coli production and purification of PASylated test proteins

The PASylated fusion proteins PAS#1(800)-IL1Ra and H1GA-PAS#1(200)-His_6 were expressed in the cytoplasm or via periplasmic secretion, respectively, and purified as previously described.[4] Yields from cytoplasmic expression were around ≥10 mg and those from periplasmic secretion ≤1 mg per liter bacterial culture. *Strep*II-eGFP-PAS#1(200) was produced as soluble protein in the cytoplasm of the *E. coli* strain BL21[5] using the generic expression vector pASK75[6] in 2 liter shake flask culture. The bacteria were lysed via sonication with a Digital Sonifier 250 (Branson, Danbury, CT) and the protein was purified by *Strep*-Tactin affinity chromatography[7] as well as size exclusion chromatography (SEC) on a Superdex200 16/60 pg column (GE Healthcare, Munich, Germany).

Coupling of the anti-PAS Fab to NHS-activated Sepharose

The purified Fab was concentrated to 2.5–5 mg/ml in 200 mM $NaHCO_3$, 500 mM NaCl, pH 8.3 (adjusted with NaOH) using an Amicon Ultra 10 kDa centrifugal filter (Millipore, Eschborn, Germany). Coupling to NHS-activated Sepharose 4 Fast Flow (GE Healthcare) was performed according to the manufacturer‘s instructions in the same buffer using 5 mg purified Fab for 2 ml resuspended bead slurry, corresponding to 1 ml settled resin, with an incubation for 3 h at room temperature. Washing of the Sepharose beads was performed by

resuspension in a 15 ml centrifuge tube and centrifugation for 1 min at 1000 rpm in a 4K centrifuge (Sigma Laborzentrifugen, Osterode, Germany) in six alternating steps, each with 6 ml of the following two buffers: (i) 100 mM Tris/HCl pH 8.0 (to inactivate excess reactive groups) and (ii) 100 mM Na-acetate, 500 mM NaCl, pH 4.0. Alternatively, coupling to a prepacked 1 ml NHS-activated HiTrap HP column (GE Healthcare) was performed, again following the manufacturer‘s instructions, using 5 mg Fab per column for a total incubation time of 90 min at room temperature. To this end, the protein solution was recirculated through the column from time to time using a pair of syringes attached to both ends. Blocking of excess reactive groups and removal of non-covalently bound protein was subsequently achieved by washing on an ÄKTA pure system (GE Healthcare) in six alternating steps, each with 6 ml of with the following buffers: (i) 500 mM ethanolamine/HCl, 500 mM NaCl, pH 8.3 and (ii) 100 mM Na-acetate, 500 mM NaCl, pH 4.0. SDS-PAGE analysis of the supernatant and column flow-through after the coupling step confirmed essentially complete immobilization of the Fab. Fab-charged beads and columns were stored at 4 °C in binding buffer supplemented with 0.1 % (w/v) NaN_3 to prevent microbial growth.

Screening for suitable elution conditions with the anti-PAS Fab chromatography matrix

Initial tests for binding of PASylated proteins and suitable elution conditions from the PAS affinity matrix were performed in 1.5 ml microcentrifuge tubes using 40 µl of a 50 % (v/v) slurry of Sepharose 4 Fast Flow beads charged with the anti-PAS Fab 1.2 as described above. Beads were first washed with binding buffer by repeated pipetting with a truncated plastic tip. After centrifugation at 2000 rpm for 1 min in a Minispin Plus tabletop microcentrifuge (Eppendorf, Hamburg, Germany) the clear supernatant was withdrawn. Then, 80 µl of 0.2 mg/ml H1GA-PAS#1(200)-His_6 in binding buffer was mixed with the beads and incubated for 1 min. After sedimentation by centrifugation, the beads were washed twice with 160 µl binding buffer each. Then, the beads were mixed with 80 µl of the respective elution buffer and incubated for 5 min, again followed by centrifugation. To avoid accidental carry-over of beads at this step, only the upper 40 µl of the clear supernatant was withdrawn

for sampling of the eluted PASylated protein via SDS-PAGE. To test whether the elution condition had affected the activity of the affinity matrix, the entire procedure was repeated in a second cycle of binding and elution, again followed by SDS-PAGE analysis.

Production of cell extracts containing PASylated test proteins

To investigate the applicability of our PAS affinity chromatography for the one-step purification of PASylated proteins from cellular extracts, total cell extract (CE) or periplasmic extract was prepared for in total three PASylated proteins. In the first case, *E. coli* BL21 was transformed with the corresponding expression plasmid for *Strep*II-eGFP-PAS#1(200) or PAS#1(800)-IL1Ra and grown at 30 °C to OD_{600} ≈ 0.5. Recombinant gene expression was induced by the addition of 0.2 µg/ml anhydrotetracycline (Acros Organics, Geel, Belgium) or 1 mM isopropyl β-D-1-thiogalactopyranoside (Formedium, Hunstanton, UK), respectively. After 3 h, the bacteria were harvested by centrifugation at 4500 rpm (Sorvall RC 12BP Plus with H-12000 rotor; Thermo Fisher Scientific, Munich, Germany) and the bacterial pellet was resuspended in binding buffer (100 mM Tris/HCl pH 8.0, 150 mM NaCl, 1 mM EDTA), followed by cell lysis via sonication as described above. Cell debris was sedimented by centrifugation for 40 min at 15.000 rpm in a Sorvall RC 6C Plus centrifuge (Thermo Fisher Scientific) and the supernatant was sterile-filtered through a 0.45 µm membrane (Sartorius, Göttingen, Germany). To prepare the periplasmic extract (PE) containing H1GA-PAS#1(200)-His_6, *E. coli* JM83 transformed with the corresponding expression plasmid[8] was grown at 22 °C to OD_{600} ≈ 0.6 and recombinant gene expression was induced for 3 h by the addition of 0.2 µg/ml anhydrotetracycline. Again, the bacteria were harvested by centrifugation at 4500 rpm and the PE was prepared by incubating the cells in a hyperosmotic buffer (100 mM Tris/HCl pH 8.0, 500 mM sucrose, 1 mM EDTA), followed by separation of the spheroplasts via centrifugation as previously described.[3b, 4b]

PAS affinity chromatography with a packed 1 ml column on an automated HPLC system

All purification runs with the 1 ml bed volume HiTrap HP column charged with the anti-PAS Fab 1.2 as described above were performed on an ÄKTA pure HPLC system (GE Healthcare) equipped with a multiwavelength detector and using 100 mM Tris/HCl pH 8.0, 150 mM NaCl, 1 mM EDTA as running buffer at a flow rate of 1 ml/min. After an initial equilibration step with 2 column volumes (CV) of running buffer, the PASylated protein sample was injected from a capillary loop, followed by column washing with running buffer. In case of the purification of *Strep*II-eGFP-PAS#1(200) or PAS#1(800)-IL1Ra from the *E. coli* whole cell extract (CE), up to 1 ml of the sterile-filtered CE was applied to the column. For the purification of H1GA-PAS#1(200)-His_6 from the periplasmic cell extract (PE), 15 ml sterile-filtered PE was applied using two 10 ml sample loops operated in series. Elution of the bound PASylated protein was achieved by applying 2–3 CV of elution buffer (1 M L-prolinamide, 100 mM Tris, 150 mM NaCl, 1 mM EDTA, pH adjusted to 8.0 with HCl). Regeneration of the column was achieved by washing with 2–3 CV of running buffer. Due to an apparent impurity in the commercial L-prolinamide substance (Carbosynth, Bratislava, Slovakia) that led to a background absorption at 280 nm, a blank chromatogram (without application of a PASylated protein) was recorded and used for subtraction to obtain the corrected chromatogram. Such a correction was not necessary when detecting the PASylated eGFP absorption at 488 nm via its fluorophore.

Protein analysis via SEC

Analytical SEC was performed on an ÄKTA pure HPLC system using a Superdex 200 10/300 GL column (GE Healthcare) with 20 mM HEPES/NaOH pH 7.5, 150 mM NaCl as running buffer at a flow rate of 0.5 ml/min. Chromatograms and retention volumes of conventionally purified *Strep*II-eGFP-PAS#1(200), PAS#1(800)-IL1Ra and H1GA-PAS#1(200)-His_6 were compared with the respective protein preparation obtained using the 1 ml PAS affinity column as described above.

Supporting References

[1] J. Schilz, U. Binder, L. Friedrich, M. Gebauer, C. Lutz, M. Schlapschy, A. Schiefner, A. Skerra, *J. Mol. Biol.* **2021**, 167113.
[2] H. J. Meerman, G. Georgiou, *Bio/technology* **1994**, *12*, 1107-1110.
[3] a) W. Schiweck, A. Skerra, *Proteins* **1995**, *23*, 561-565; b) A. Skerra, *Gene* **1994**, *141*, 79-84.
[4] a) N. E. Powers, B. Swartzwelter, C. Marchetti, D. M. de Graaf, A. Lerchner, M. Schlapschy, R. Datar, U. Binder, C. K. Edwards, 3rd, A. Skerra, C. A. Dinarello, *J. Biol. Chem.* **2020**, *295*, 868-882; b) S. Rauth, D. Hinz, M. Börger, M. Uhrig, M. Mayhaus, M. Riemenschneider, A. Skerra, *Biochem. J.* **2016**, *473*, 1563-1578.
[5] P. Daegelen, F. W. Studier, R. E. Lenski, S. Cure, J. F. Kim, *J. Mol. Biol.* **2009**, *394*, 634-643.
[6] A. Skerra, *Gene* **1994**, *151*, 131-135.
[7] T. G. M. Schmidt, A. Eichinger, M. Schneider, L. Bonet, U. Carl, D. Karthaus, I. Theobald, A. Skerra, *J. Mol. Biol.* **2021**, *433*, 166893.
[8] C. Yanisch-Perron, J. Vieira, J. Messing, *Gene* **1985**, *33*, 103-119.
[9] a) J. Breibeck, A. Skerra, *Biopolymers* **2018**, *109*, e23069; b) M. Schlapschy, U. Binder, C. Börger, I. Theobald, K. Wachinger, S. Kisling, D. Haller, A. Skerra, *Protein Eng., Des. Sel.* **2013**, *26*, 489-501.

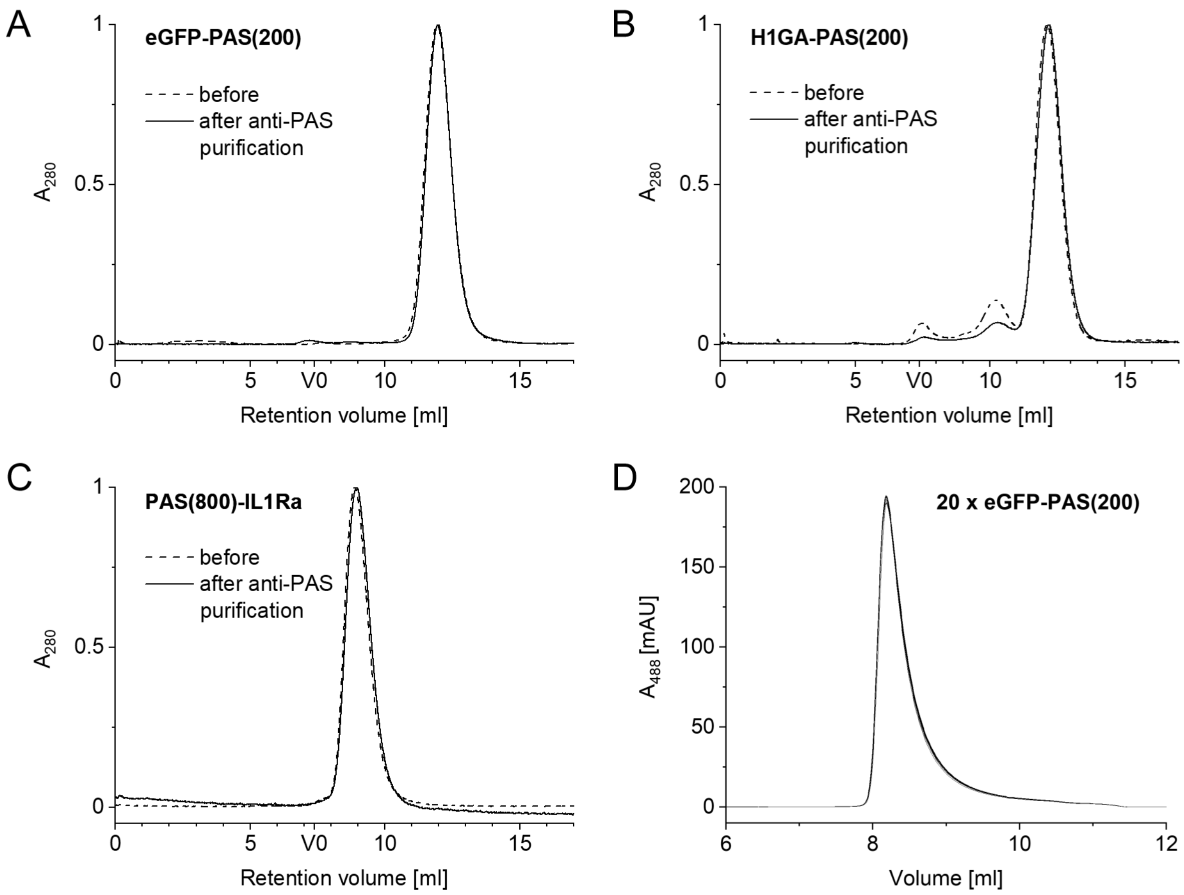

Figure S1: Analytical SEC of purified *Strep*II-eGFP-PAS#1(200) (A), H1GA-PAS#1(200)-His_6 (B) and PAS#1(800)-IL1Ra (C) before (dashed line) and after (solid line) one cycle of PAS affinity chromatography. (D) Superposition of chromatograms from 20 repeated PAS affinity purification runs of pre-purified *Strep*II-eGFP-PAS#1(200) on a 1 ml PAS affinity column monitored at a wavelength of 488 nm, indicative of the eGFP fluorophore.

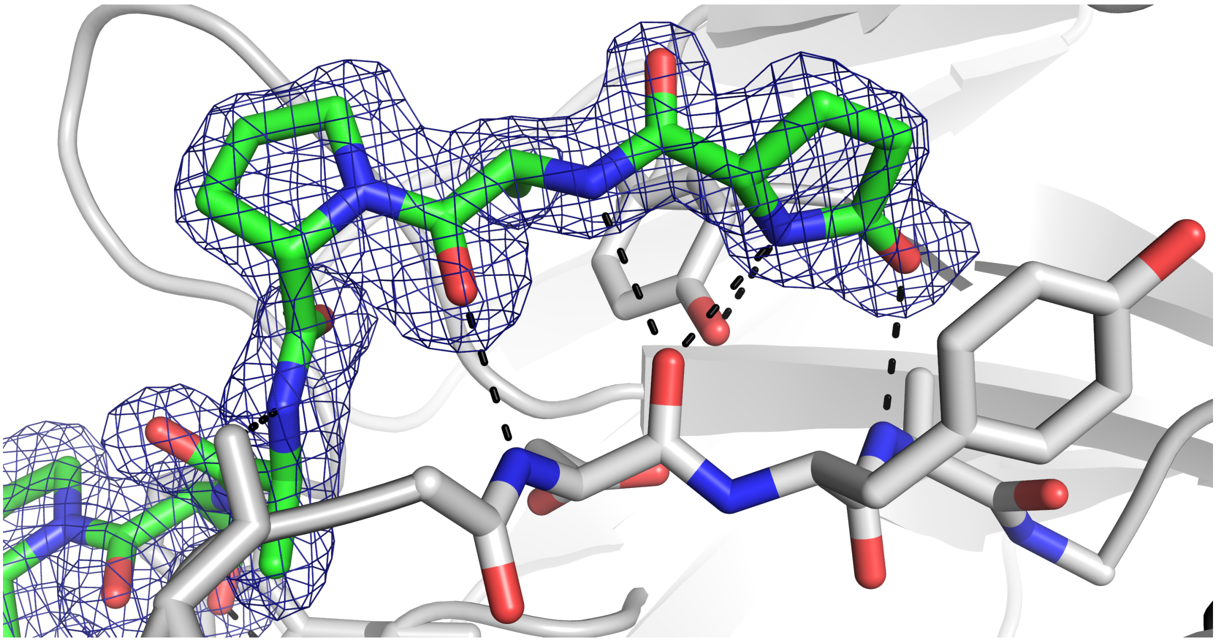

Figure S2: Detailed interactions between the Fab 1.2 (grey) and the bound PAS#1 epitope peptide (green), including the N-terminal pyroglutamyl group of the peptide that was used for co-crystallization. The $2F_O$-F_C electron density map is displayed at a contour level of 1σ (PDB ID: 7O31). Hydrogen bonds are indicated by black dashes.

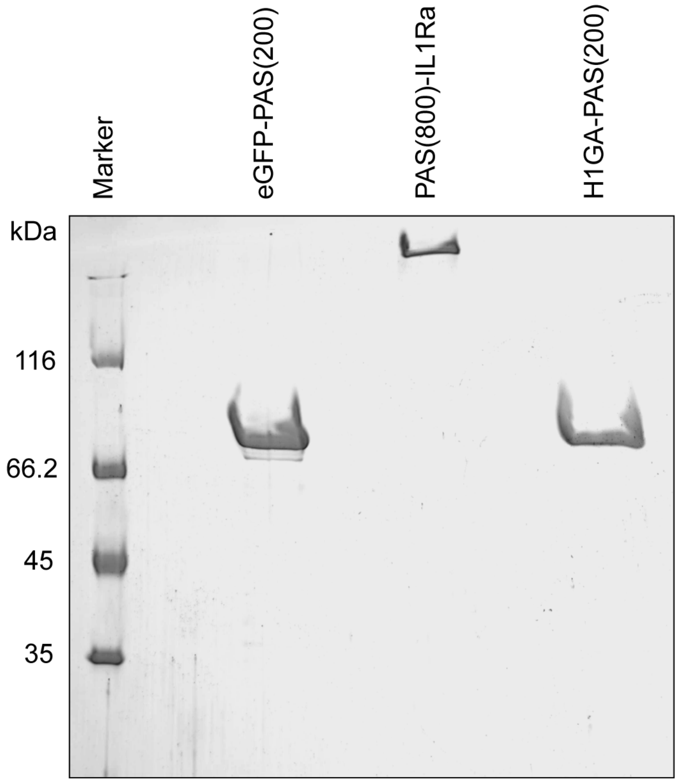

Figure S3: Coomassie brilliant blue stained SDS-PAGE showing *Strep*II-eGFP-PAS#1(200), PAS#1(800)-IL1Ra and H1GA-PAS#1(200)-His$_6$ directly purified from the whole cell extract or periplasmic extract of *E. coli* by one-step PAS affinity purification and elution with L-prolinamide using an elevated sample load of 26 µg, 85 µg and 10 µg protein, respectively. Please note that PASylated proteins get poorly stained with Coomassie brilliant blue with increasing length of the PAS tag.[9]

Scheme S1: Amino acid sequences of proteins used in this work (PAS sequences bold).

Anti-PAS Fab 1.2 – heavy chain:

EVQLVESGGGLVQPGGSLKLSCAASGFTFSSYGMSWVRQTPDKRLELVATINSNGGSTYYLDSVKGRFTISRDKA
KNTLYLQMSSLKSEDTAMYYCVRGGSIYDGYDYAMDYWGQGTSVTVSSASTKGPSVFPLAPSSKSTSGGTAALGC
LVKDYFPEPVTVSWNSGALTSGVHTFPAVLQSSGLYSLSSVVTVPSSSLGTQTYICNVNHKPSNTKVDKKVEPKS
CHHHHHH

Anti-PAS Fab 1.2 – light chain:

DIVLTQSPASLAVSLGQRATISCRASQSVSTSSYSYMHWFQQKPGQPPKLLIKYASNLESGVPARFSGSGSGTDF
TLNIHPVEEEDTATYYCQHSWEIPLTFGAGTKLEIKRTVAAPSVFIFPPSDEQLKSGTASVVCLLNNFYPREAKV
QWKVDNALQSGNSQESVTEQDSKDSTYSLSSTLTLSKADYEKHKVYACEVTHQGLSSPVTKSFNRGEC

*Strep*II-eGFP-PAS#1(200):

ASWSHPQFEKGASKGEELFTGVVPILVELDGDVNGHKFSVSGEGEGDATYGKLTLKFICTTGKLPVPWPTLVTTL
TYGVQCFSRYPDHMKRHDFFKSAMPEGYVQERTIFFKDDGNYKTRAEVKFEGDTLVNRIELKGIDFKEDGNILGH
KLEYNYNSHNVYIMADKQKNGIKVNFKIRHNIEDGSVQLADHYQQNTPIGDGPVLLPDNHYLSTQSALSKDPNEK
RDHMVLLEFVTAAGITHGMDELYQ**ASPAAPAPASPAAPAPSAPAASPAAPAPASPAAPAPSAPAASPAAPAPASP**
AAPAPSAPAASPAAPAPASPAAPAPSAPAASPAAPAPASPAAPAPSAPAASPAAPAPASPAAPAPSAPAASPAAP
APASPAAPAPSAPAASPAAPAPASPAAPAPSAPAASPAAPAPASPAAPAPSAPAASPAAPAPASPAAPAPSAPAA

H1GA-PAS#1(200)-His$_6$:

QDSTSDLIPAPPLSKVPLQQNFQDNQFHGKWYVVGAAGNVLLREDKDPLKMYATIYELKEDKSYNVTSVGFDDKK
CLYKIRTFVPGSQPGEFTLGRIKSEPGGTSWLVRVVSTNYNQHAMVFFKEVAQNRETFNITLYGRTKELTSELKE
NFIRFSKSLGLPENHIVFPVPIDQCIDGSS**ASPAAPAPASPAAPAPSAPAASPAAPAPASPAAPAPSAPAASPAA**
PAPASPAAPAPSAPAASPAAPAPASPAAPAPSAPAASPAAPAPASPAAPAPSAPAASPAAPAPASPAAPAPSAPA
ASPAAPAPASPAAPAPSAPAASPAAPAPASPAAPAPSAPAASPAAPAPASPAAPAPSAPAASPAAPAPASPAAPA
PSAPASAHHHHHH

PAS#1(800)-IL1Ra:

PASPAAPAPASPAAPAPSAPAASPAAPAPASPAAPAPSAPAASPAAPAPASPAAPAPSAPAASPAAPAPASPAAP
APSAPAASPAAPAPASPAAPAPSAPAASPAAPAPASPAAPAPSAPAASPAAPAPASPAAPAPSAPAASPAAPAPA
SPAAPAPSAPAASPAAPAPASPAAPAPSAPAASPAAPAPASPAAPAPSAPAASPAAPAPASPAAPAPSAPAASPA
APAPASPAAPAPSAPAASPAAPAPASPAAPAPSAPAASPAAPAPASPAAPAPSAPAASPAAPAPASPAAPAPSAP
AASPAAPAPASPAAPAPSAPAASPAAPAPASPAAPAPSAPAASPAAPAPASPAAPAPSAPAASPAAPAPASPAAP
APSAPAASPAAPAPASPAAPAPSAPAASPAAPAPASPAAPAPSAPAASPAAPAPASPAAPAPSAPAASPAAPAPA
SPAAPAPSAPAASPAAPAPASPAAPAPSAPAASPAAPAPASPAAPAPSAPAASPAAPAPASPAAPAPSAPAASPA
APAPASPAAPAPSAPAASPAAPAPASPAAPAPSAPAASPAAPAPASPAAPAPSAPAASPAAPAPASPAAPAPSAP
AASPAAPAPASPAAPAPSAPAASPAAPAPASPAAPAPSAPAASPAAPAPASPAAPAPSAPAASPAAPAPASPAAP
APSAPAASPAAPAPASPAAPAPSAPAASPAAPAPASPAAPAPSAPAASPAAPAPASPAAPAPSAPAASPAAPAPA
SPAAPAPSAPAASPAAPAPASPAAPAPSAPAASPAAPAPASPAAPAPSAPAARPSGRKSSKMQAFRIWDVNQKTF
YLRNNQLVAGYLQGPNVNLEEKIDVVPIEPHALFLGIHGGKMCLSCVKSGDETRLQLEAVNITDLSENRKQDKRF
AFIRSDSGPTTSFESAACPGWFLCTAMEADQPVSLTNMPDEGVMVTKFYFQEDE
